# 165. Real-world Abrysvo Vaccine Effectiveness (VE) against Respiratory Syncytial Virus (RSV)-related Severe Acute Respiratory Infection (ARI) Hospitalizations and Emergency Department (ED) Visits—Kaiser Permanente of Southern California (KPSC), November 2023–April 2024

**DOI:** 10.1093/ofid/ofae631.002

**Published:** 2025-01-29

**Authors:** Sara Y Tartof, Negar Aliabadi, Gabriella Goodwin, Jeff Slezak, Vennis Hong, Bradley Ackerson, Bradley Ackerson, Qing Liu, Sally Shaw, Sabrina Welsh, Julie Stern, Banshri Kapadia, Brigitte Spence, Joe Lewnard, Gregory Davis, Michael Aragones, Michael Dutro, Erica Chilson, Elisa Gonzalez, Robin Hubler, Ashley Miller, Brandon Chia, Luis Jodar, Bradford D Gessner, Elizabeth Begier

**Affiliations:** Kaiser Permanente Southern California, Pasadena, CA; Pfizer, New York, New York; Kaiser Permanente, Southern California, Pasadena, California; Kaiser Permanente Southern California, Pasadena, CA; Kaiser Permanente, El Monte, California; Kaiser Permanente Southern California, Pasadena, CA; Kaiser Permanente Southern California, Pasadena, CA; Pfizer Inc., Collegeville, Pennsylvania; Kaiser Permanente Southern California, Pasadena, CA; Pfizer, Inc, Collegeville, Pennsylvania; Kaiser Permanente Southern California, Pasadena, CA; Kaiser Permanente, Southern California, Pasadena, California; Kaiser Permanente Southern California, Pasadena, CA; University of California, Berkeley, San Francisco, California; Kaiser Permanente, Southern California, Pasadena, California; Kaiser Permanente Southern California, Pasadena, CA; Pfizer, New York, New York; Pfizer, New York, New York; Pfizer, New York, New York; Pfizer Inc., Collegeville, Pennsylvania; Pfizer, New York, New York; Kaiser Permanente School of Medicine, South Pasadena, California; Pfizer Vaccines, Collegeville, Pennsylvania; Pfizer Biopharma Group, Collegeville, Pennsylvania; Pfizer Vaccines, Collegeville, Pennsylvania

## Abstract

**Background:**

Abrysvo’s pivotal trial analysis of efficacy against severe RSV-related respiratory disease was hampered by accrual of few hospitalizations/ED events. In this post-licensure study, we evaluated Abrysvo VE against 1st RSV-related severe ARI in KPSC, a large healthcare system.

**Figure:**
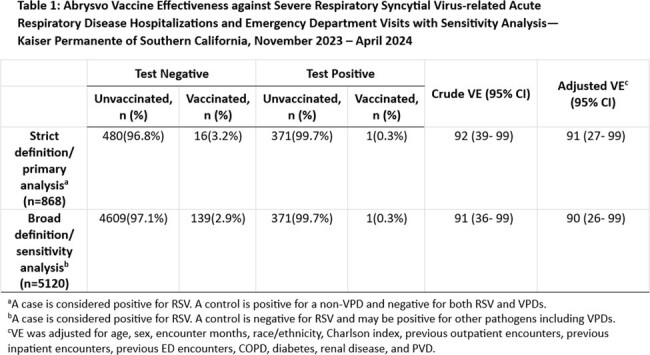

**Methods:**

We assessed Abrysvo VE among adults aged ≥ 60 years through KPSC members’ electronic health records plus enhanced specimen testing using a test-negative case-control study (study period: 11/24/2023–4/9/2024). ARI was defined as ED/hospitalizations with an ARI ICD-10 code, with respiratory specimens tested on GenMark RP2 expanded multiplex PCR assay; severe ARI required supplemental oxygen use. Cases were defined as RSV+ ARIs. Two sets of pre-specified controls were used: ‘Strict’: RSV- and positive for a non-vaccine preventable disease (VPD), and ‘Broad’: all RSV-. The strict group accounted for lower sensitivity of RSV testing in adults and potential bias with VPD controls. Exposure was Abrysvo receipt ≥ 21 days before encounter. Odds ratios (OR) and 95% confidence intervals (CI) were estimated from multivariable logistic regression adjusted for demographic and clinical characteristics. VE (1−OR X 100%) was estimated for severe ARI, ARI hospitalization/ED visit (separately and combined), and ARI among those with high-risk conditions.

**Figure:**
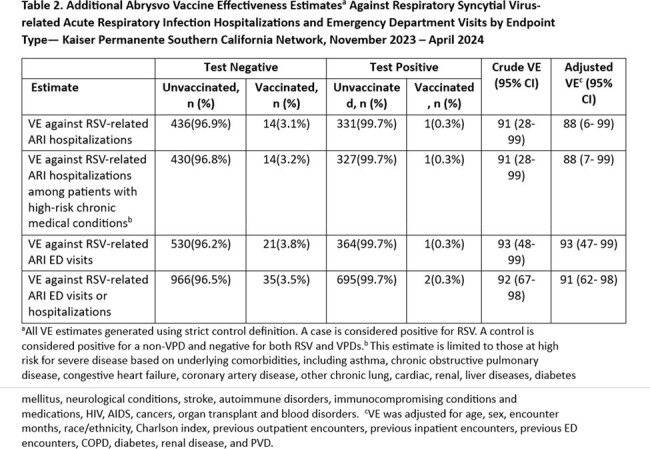

**Results:**

Among 5,120 KPSC members in the study population, > 50% were ≥ 75 years, 95% had ≥ 1 underlying comorbidities, 15% were immunocompromised. For RSV-related severe ARI (Table 1), the strict analysis included 372 cases and 496 controls; the vaccinated received Abrysvo a median of 69 days before the ARI. Adjusted VE was 91% (95% CI: 27–99). The broad analysis included 372 cases and 4,748 controls; the vaccinated received Abrysvo a median of 62.5 days before the ARI. Adjusted VE was 90% (95% CI: 26–99). Additional VE estimates ranged from 88–91% among different subsets of the ARI hospitalization/ED visit population (Table 2).

**Conclusion:**

This study demonstrated real-world Abrysvo VE during its first 5 months of use against severe ARI in hospital/ED settings among US adults ≥ 60 years of age. These results expand on clinical trial results by including severe disease endpoints and a substantial proportion of high-risk persons.

**Disclosures:**

**Sara Y. Tartof, PhD MPH**, GSK: Funding paid directly to institution; vaccines provided directly to institution|Pfizer Inc.: Funding paid directly to institution; vaccines provided directly to institution **Negar Aliabadi, MD, MS**, Pfizer: I am an employee of Pfizer and own stocks in the company **Jeff Slezak, MS**, Dynavax Technologies: Grant/Research Support|Pfizer, Inc.: Grant/Research Support **Qing Liu, M.S.**, Pfizer Inc: Stocks/Bonds (Public Company) **Michael Dutro, PharmD**, Pfizer: Employee|Pfizer: Stocks/Bonds (Public Company) **Erica Chilson, PharmD**, Pfizer, Inc: Employee|Pfizer, Inc: Stocks/Bonds (Public Company) **Elisa Gonzalez, MS**, Pfizer: Stocks/Bonds (Private Company) **Ashley Miller, MA, CCRP**, Pfizer: Stocks/Bonds (Public Company) **Bradford D. Gessner, M.D., M.P.H.**, Pfizer: Employee|Pfizer: Stocks/Bonds (Public Company) **Elizabeth Begier, MD, M.P.H.**, Pfizer: Employee|Pfizer: Stocks/Bonds (Public Company)

